# Coordinated Cell-Wall and Starch Maturation Is Associated with Winter-Harvest Quality in *Sparganium stoloniferum* Tubers

**DOI:** 10.3390/ijms27104566

**Published:** 2026-05-19

**Authors:** Xilong Qian, Maoqi Pan, Jingying Zhang, Qinan Liu, Fan Yang, Chanchan Liu, Mengru Sang, Qinan Wu

**Affiliations:** 1 National Key Laboratory on Technologies for Chinese Medicine Pharmaceutical Process Control and Intelligent Manufacture, Nanjing University of Chinese Medicine, Nanjing 210023, China; qxl@njucm.edu.cn (X.Q.); 300586@njucm.edu.cn (F.Y.); liuchanchan@njucm.edu.cn (C.L.); 2 Jiangsu Collaborative Innovation Center of Chinese Medicinal Resources Industrialization, Nanjing University of Chinese Medicine, Nanjing 210023, China; 3 School of Pharmacy, Nanjing University of Chinese Medicine, Nanjing 210023, China; pan12@njucm.edu.cn (M.P.); zjy20030823@163.com (J.Z.); 4 Nanjing Institute for Food and Drug Control, Nanjing 211198, China; liuqinan0728@163.com

**Keywords:** *Sparganium stoloniferum* tubers (SL), winter-harvest quality, firm texture, cell wall, starch, transcriptome

## Abstract

*Sparganium stoloniferum* tubers (SL), known medicinally as Sparganii Rhizoma, are commonly considered superior at the winter-harvest stage, when they show the traditional quality traits of heavy weight and firm texture. However, the developmental basis of this quality phenotype remains insufficiently understood. This study aimed to determine how tissue organization, cell-wall architecture, starch deposition, and related transcriptional patterns are associated with winter-harvest quality in SL. By comparing SL at different developmental stages, we found that maturation was accompanied by reduced moisture content, increased tuber density, higher parenchyma cell density, progressive cell-wall thickening, and marked starch accumulation. Laser scanning confocal microscopy (LSCM), scanning electron microscopy (SEM), and transmission electron microscopy (TEM) observations further revealed thickened multilamellar cell walls and abundant clustered or compound-like starch bodies in mature SL. Starch isolated from mature SL displayed an A-type crystalline pattern, short-range order, and high gelatinization and pasting temperatures, indicating an ordered and thermally stable starch matrix. Cell-wall Fourier-transform infrared spectroscopy (FTIR) and solid-state nuclear magnetic resonance (NMR) analyses showed a predominantly polysaccharide-rich framework with subtle maturation-associated changes in aromatic- and methoxy-associated wall signals. Transcript-guided pathway analysis, supported by reverse transcription quantitative polymerase chain reaction (RT–qPCR)validation, suggested developmental shifts in carbohydrate metabolism, lipid-related metabolism, and gibberellin-associated transcriptional patterns. Together, these findings indicate that winter-harvest quality in SL is associated with coordinated tissue consolidation, cell-wall maturation, starch deposition, and transcriptional reprogramming, providing a structural and molecular framework for understanding the traditional firm-texture trait of *S. stoloniferum*.

## 1. Introduction

Underground storage organs are major sink tissues in which assimilated carbon is imported, allocated, and converted into structural and reserve components during development. This process is reflected not only in biomass accumulation, but also in quality-related traits such as organ size, dry-matter accumulation, tissue density, storage-reserve deposition, and texture. In cassava, predominantly symplastic phloem unloading into storage roots is closely associated with efficient starch accumulation [1], while source–sink coordination in sweet potato integrates assimilate production, transport, unloading, and allocation to determine starch yield [2]. Similar developmental links among starch and sucrose metabolism, cell-wall-related processes, and hormone-associated pathways have been reported in the medicinal plant *Callerya speciosa* [3]. These studies suggest that the quality of underground storage organs is often shaped by coordinated tissue filling, reserve deposition, and developmental regulation, although how these processes relate to traditional quality traits remains insufficiently understood in many medicinal organs.

*Sparganium stoloniferum* is a medicinal plant widely distributed in Asia and has long been used in Asian medicine [4,5]. *S. stoloniferum* tubers (SL), known medicinally as Sparganii Rhizoma, are used in traditional Chinese medicine for promoting blood circulation, removing blood stasis, and alleviating mass-related disorders [6]. In traditional quality evaluation, SL are generally regarded as superior when they are heavy and firm in texture, and this macroscopic trait remains an important criterion in medicinal identification and quality assessment [7]. In the present study, winter-harvest quality refers primarily to these traditional mature-tuber traits, together with associated measurable features observed during maturation, including increased tuber density, reduced moisture content, greater tissue compactness, cell-wall thickening, and starch accumulation. Previous work using the same developmental sample set showed that SL increased in size, weight, and rigidity from June to December [8], but the anatomical, cellular, and biochemical features underlying this quality phenotype remain unclear.

Plant tissue firmness is strongly influenced by both cell-wall architecture and intracellular storage deposition. In fleshy organs, pectin depolymerization and wall disassembly reduce tissue firmness, whereas maintenance of wall integrity and intercellular adhesion contributes to stronger tissue mechanics [9,10]. In storage organs, starch deposition may further promote tissue filling and consolidation, while starch functional properties are affected by its molecular and supramolecular organization. Starch–lipid interactions can influence starch structure, thermal stability, and pasting-related properties [11,12,13], suggesting that starch in medicinal underground organs should be considered not only in terms of amount, but also in terms of structural state. Developmental regulators may coordinate these processes. For example, hormonal pathways, including gibberellin-associated pathways, can influence sink activity, starch synthesis, secondary growth, and storage-organ development in several plant systems [14,15]. However, the relationship between such regulatory processes and the formation of traditional quality traits in medicinal underground organs remains largely unresolved.

Despite the longstanding medicinal use of *S. stoloniferum*, an integrated framework linking tissue organization, starch properties, cell-wall chemistry, and associated transcriptional changes is still lacking. Winter-harvest SL represents the mature stage at which the characteristic heavy and firm quality trait becomes most pronounced, making it a suitable system for examining how anatomical and metabolic changes are associated with medicinal quality. In the present study, we investigated the developmental basis of winter-harvest quality in SL by integrating histological observation, ultrastructural analysis, starch physicochemical characterization, cell-wall spectroscopic analysis, and transcript-guided pathway analysis. We aimed to determine how changes in tissue organization, starch properties, cell-wall chemistry, and associated transcriptional patterns are related to the characteristic firm texture of winter-harvest SL.

## 2. Results

### 2.1. Developmental Changes in Tissue Organization, Cell-Wall Thickness, Starch Accumulation, and Apparent Amylose Content

**Figure 1:**
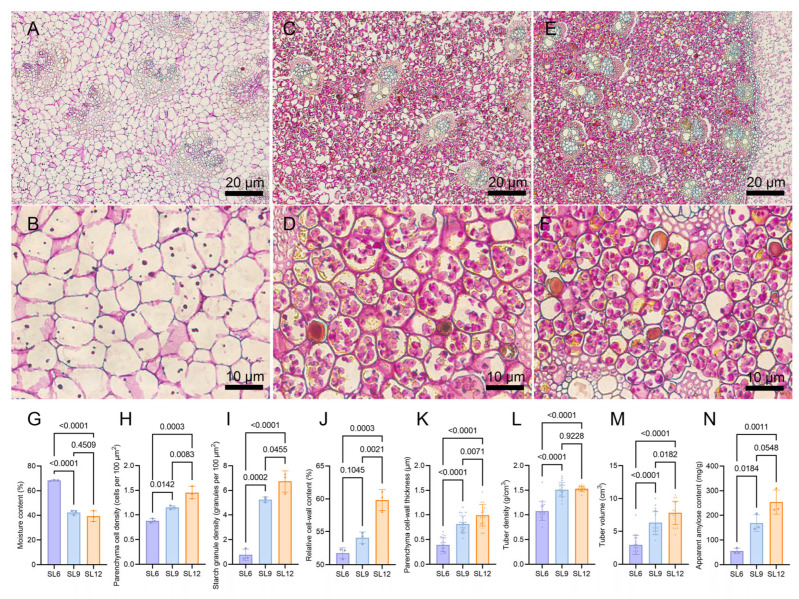
PAS–Naphthol Yellow S staining and quantitative analysis of starch- and cell-wall-related traits in *Sparganium stoloniferum* tubers (SL) at different developmental stages. (**A**,**B**) Tuber tissues collected in June. (**C**,**D**) Tuber tissues collected in September. (**E**,**F**) Tuber tissues collected in December. (**G**) Moisture content. (**H**) Parenchyma cell density. (**I**) Starch granule density. (**J**) Relative cell-wall content. (**K**) Parenchyma cell-wall thickness. (**L**) Tuber density. (**M**) Tuber volume. (**N**) Apparent amylose content. SL6, SL9, and SL12 represent tubers collected in June, September, and December, respectively. Data are presented as mean ± SD, with dots representing individual measurements. For panels (**G**–**J**,**N**), *n* = 3 biological replicates. For panels (**K**–**M**), *n* = 20 independent tubers. Statistical significance was assessed by one-way ANOVA followed by Tukey’s multiple-comparisons test, and exact *p* values are shown.

PAS–Naphthol Yellow S staining revealed marked developmental changes in the internal structure of SL from June to December (Figure 1A–F). At the June stage, the tubers (SL6) were characterized by relatively large and loosely arranged parenchyma cells with sparse intracellular granules and faintly stained cell walls. At the September stage, the tissues (SL9) became more compact, accompanied by increased parenchyma cell density, more abundant intracellular starch granules, and stronger staining of wall regions. By the December harvest stage, the tuber tissues (SL12) appeared densely packed and markedly enriched in intracellular contents, with abundant starch granules and visibly thickened parenchyma cell walls. Quantitative analysis further supported these observations. Moisture content decreased significantly from SL6 to SL9 and remained lower at SL12 (Figure 1G), whereas parenchyma cell density, starch granule density, relative cell-wall content, and parenchyma cell-wall thickness all increased significantly during development (Figure 1H–K). Tuber density also increased significantly from SL6 to SL9 and remained high at SL12 (Figure 1L), while tuber volume increased progressively across the three developmental stages (Figure 1M). Apparent amylose content also increased during development, with significantly higher levels in SL9 and SL12 than in SL6 (Figure 1N). The difference between SL9 and SL12 showed an increasing trend but did not reach statistical significance. Together, these results show that SL maturation is accompanied by reduced moisture content, increased tissue compactness and density, thicker parenchyma cell walls, greater starch accumulation, and increased apparent amylose content.

### 2.2. Confocal Visualization of Cell-Wall Organization and Intracellular Starch Deposition in Winter-Harvest SL

**Figure 2:**
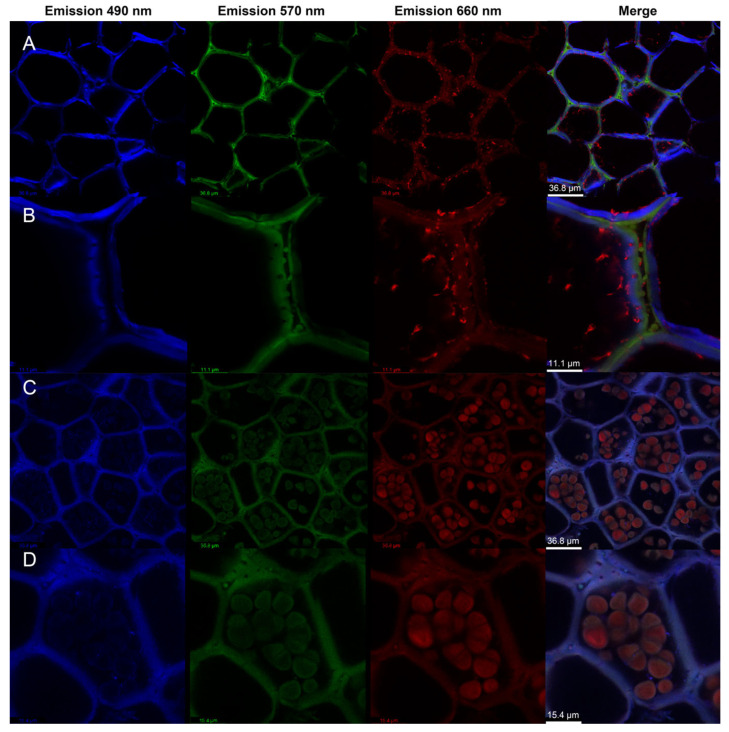
Confocal imaging of cell-wall organization and intracellular starch deposition in mature *Sparganium stoloniferum* tubers (SL12). (**A**,**B**) Calcofluor white-stained sections showing parenchyma cell walls with distinct wall organization and thickening at different magnifications. (**C**,**D**) Hydroxynaphthol blue-stained sections showing abundant intracellular starch granules in parenchyma cells at different magnifications. Fluorescence signals were collected at emission wavelengths of 490, 570, and 660 nm, and merged images are shown in the rightmost column. Scale bars: (**A**,**C**), 36.8 μm; (**B**), 11.1 μm; (**D**), 15.4 μm.

Laser scanning confocal microscopy (LSCM) provided further visualization of the subcellular organization of mature tuber parenchyma. In calcofluor white-stained sections (Figure 2A,B), fluorescence clearly outlined the parenchyma cell walls and highlighted the wall architecture at different magnifications. The wall-associated signals revealed distinct inner and outer wall regions and supported pronounced wall thickening and layered wall organization. At higher magnification, the walls exhibited obvious inward thickening and a multilayered appearance. In hydroxynaphthol blue-stained sections (Figure 2C,D), strong intracellular granular signals were observed within parenchyma cells, indicating abundant starch accumulation. The granules were relatively large and mainly occurred as compound-like starch granules with visible lamellar features. These confocal observations showed thickened wall regions and abundant intracellular starch signals in winter-harvest SL.

### 2.3. Ultrastructural Characterization of Multilamellar Cell Walls and Starch Bodies in Winter-Harvest SL

**Figure 3:**
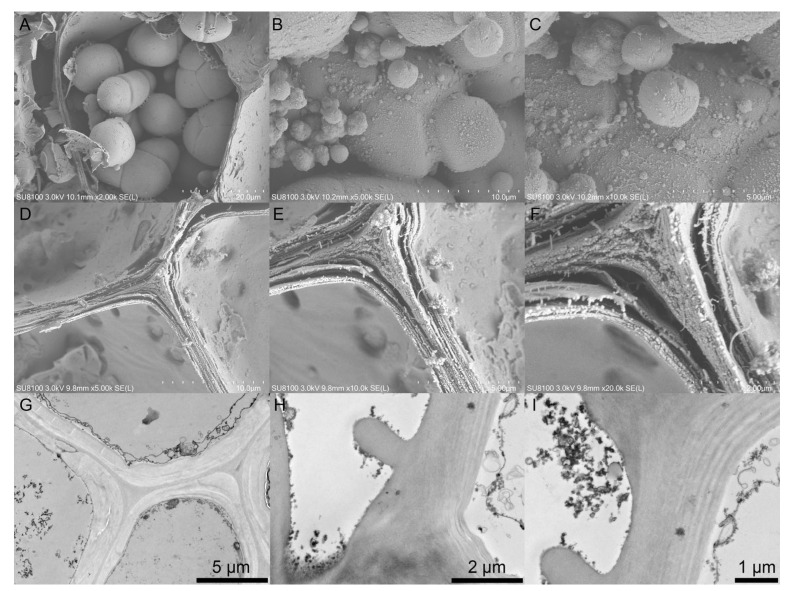
Ultrastructural characterization of starch granules and cell walls in mature *Sparganium stoloniferum* tubers (SL12). (**A**–**C**) Scanning electron microscopy (SEM) images showing abundant starch granules in parenchyma cells at different magnifications. (**D**–**F**) SEM images showing thickened and layered parenchyma cell walls at different magnifications. (**G**–**I**) Transmission electron microscopy (TEM) images showing the multilamellar architecture and inward stratified thickening of parenchyma secondary cell walls. Scale bars: (**A**), 20 μm; (**B**,**D**), 10 μm; (**C**,**E**,**G**), 5 μm; (**F**,**H**), 2 μm; (**I**), 1 μm.

Ultrastructural observations further characterized the storage and wall architecture of winter-harvest SL (Figure 3). Scanning electron microscopy (SEM) images showed that parenchyma cells were densely packed and filled with abundant starch granules (Figure 3A–C). These granules were predominantly oval to spherical, varied in size, and occurred mainly as compound-like starch granules. At higher magnification, the granule surfaces displayed membrane-like features, suggesting structural organization around individual granule units. Transmission electron microscopy (TEM) observation of starch-rich parenchyma cells showed large and angular starch-body profiles in ultrathin sections (Figure S1). The amyloplast envelope was not continuously preserved around all adjacent starch profiles. Therefore, these starch bodies are described as compound-like starch granules in a morphological sense. Examination of fractured tissues by SEM showed that parenchyma cells were tightly and uniformly arranged, forming a compact polyhedral packing pattern (Figure 3D–F). Their cell walls exhibited conspicuous thickening and a distinct layered organization. TEM analysis provided further confirmation of this architecture, showing multilamellar wall layers with inward stratified thickening surrounding the parenchyma cells (Figure 3G–I). In some regions, the secondary-wall thickness reached approximately 2 μm. Overall, the ultrastructural observations demonstrated dense starch accumulation and multilayered cell-wall thickening in winter-harvest SL.

### 2.4. Physicochemical Characterization of Starch Isolated from Winter-Harvest SL

**Figure 4:**
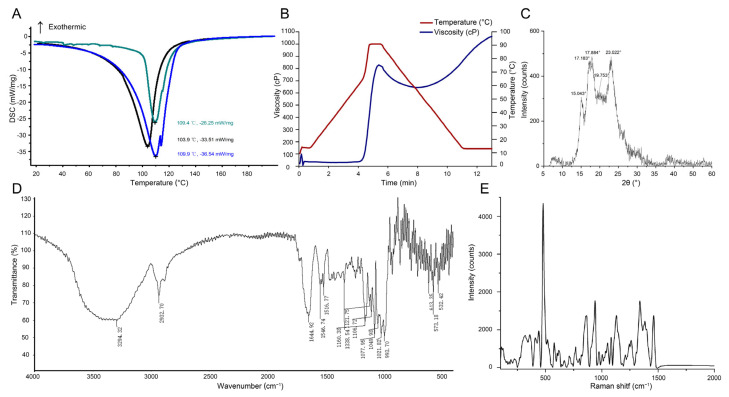
Physicochemical characterization of starch isolated from mature *Sparganium stoloniferum* tubers (SL12). (**A**) Differential scanning calorimetry (DSC) thermogram of SL12 starch. (**B**) Rapid visco analyzer (RVA) pasting profile of SL12 starch. (**C**) X-ray diffraction (XRD) pattern of SL12 starch. (**D**) Fourier transform infrared (FTIR) spectrum of SL12 starch. (**E**) Raman spectrum of SL12 starch. Numerical parameters derived from these analyses are described in the main text.

To further characterize the physicochemical properties of starch accumulated in winter-harvest SL, SL12 starch was analyzed by differential scanning calorimetry (DSC), rapid visco analysis (RVA), X-ray diffraction (XRD), Fourier transform infrared spectroscopy (FTIR), and Raman spectroscopy (Figure 4). XRD analysis showed characteristic diffraction peaks at approximately 15°, 17°, 18°, and 23°, indicating an A-type crystalline pattern, with an additional signal near 20°. The relative crystallinity was 36.03 ± 2.88%. DSC analysis revealed relatively high gelatinization temperatures, with onset temperature (T_o_), peak temperature (T_p_), and conclusion temperature (T_c_) values of 92.47 ± 8.26 °C, 107.73 ± 3.32 °C, and 118.27 ± 4.51 °C, respectively, and a gelatinization enthalpy (ΔH) of 17.77 ± 6.86 J/g. RVA showed a high pasting temperature of 91.28–91.30 °C, together with moderate peak, trough, and final viscosities. FTIR and Raman spectra displayed typical starch-associated signals, and the IR_1047/1022_ and IR_1022/995_ ratios were 0.8399 ± 0.0012 and 0.9664 ± 0.0002, respectively. The full width at half maximum of the 480 cm^−1^ band was 18.21 ± 1.10. Together, these results indicate that SL12 starch has an A-type crystalline pattern, detectable short-range molecular order, and relatively high gelatinization and pasting temperatures.

### 2.5. FTIR and Solid-State NMR Profiles of Isolated Cell-Wall Fractions

**Figure 5:**
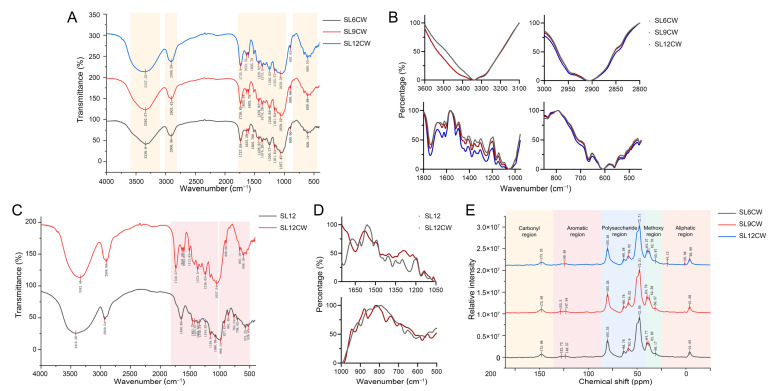
FTIR and solid-state ^13^C CP/MAS NMR analyses reveal conserved but subtly remodeled cell-wall chemistry during tuber maturation in *Sparganium stoloniferum* tubers (SL). (**A**) Overlaid FTIR spectra of cell-wall fractions (CW) isolated from SL6, SL9, and SL12. Shaded areas indicate the major absorption regions. (**B**) Normalized enlarged FTIR profiles of selected spectral regions showing subtle stage-dependent differences around 3600–3100, 3000–2800, 1800–1000, and 900–450 cm^−1^. (**C**) Comparison of FTIR spectra between whole medicinal material (SL12) and the corresponding cell-wall fractions (SL12CW). The shaded region highlights the main range in which spectral differences are evident. (**D**) Normalized enlarged views of the FTIR spectra in panel C, showing the fingerprint regions with the clearest differences between SL12 and SL12CW. (**E**) Overlaid solid-state ^13^C CP/MAS NMR spectra of CW from SL6, SL9, and SL12. Major chemical-shift regions are labeled as carbonyl, aromatic, polysaccharide, methoxy, and aliphatic regions.

FTIR spectroscopy was used to compare cell-wall fractions (CW) isolated from SL6, SL9, and SL12 (Figure 5A,B). The three CW spectra showed broadly similar overall profiles, with major absorption regions at 3600–3100, 3000–2800, 1800–1000, and 900–450 cm^−1^. A broad band around 3340 cm^−1^, a band near 2905 cm^−1^, and strong signals in the 1200–900 cm^−1^ region were detected in all samples, corresponding mainly to hydroxyl, aliphatic C–H, and carbohydrate-associated vibrations, respectively. These conserved features indicate that the isolated CW fractions retained a broadly similar polysaccharide-rich framework during tuber development. Nevertheless, normalized enlarged spectra revealed modest developmental differences, mainly in the O–H stretching envelope and the 1800–1000 cm^−1^ fingerprint region, especially around 1630, 1510–1460, 1374, 1240, 1150, 1100, and 1047 cm^−1^. Minor local differences were also visible in the 900–450 cm^−1^ region. Overall, these FTIR results suggest that SL cell walls retained similar major chemical features while undergoing subtle maturation-associated remodeling in selected spectral regions. To further distinguish wall-associated signals from those of the whole medicinal material, the FTIR spectrum of SL12CW was compared with that of the corresponding whole-material SL12 sample (Figure 5C,D). Although the two spectra shared a similar overall profile, SL12CW showed clearer local differences in the fingerprint region, especially within the 1750–1500, 1475–1300 and 1000–500 cm^−1^. These differences suggest enrichment of wall-associated spectral features after cell-wall isolation, while the overall similarity between SL12 and SL12CW reflects the carbohydrate-rich nature of the mature tuber material.

Solid-state ^13^C CP/MAS NMR provided complementary information on the chemical features of the isolated CW fractions (Figure 5E). In all three developmental stages, the spectra were dominated by resonances in the 60–110 ppm region, confirming that polysaccharides constituted the principal framework of the isolated cell walls. Signals around 100–105, 70–80, 84–90, and 60–65 ppm were assigned to anomeric carbons, polysaccharide ring carbons, cellulose-like C4 carbons, and C6-associated carbons, respectively. Weaker carbonyl, aromatic, methoxy, and aliphatic signals were also detected, and SL12CW showed slightly more prominent aromatic- and methoxy-associated signals than the earlier stages. Together, the FTIR and NMR profiles indicate that SL cell walls remain predominantly polysaccharide-rich during maturation, while subtle aromatic- and methoxy-associated wall signals become more evident in winter-harvest SL.

### 2.6. Transcript-Level Changes in Starch and Sucrose Metabolism During Tuber Maturation

**Figure 6:**
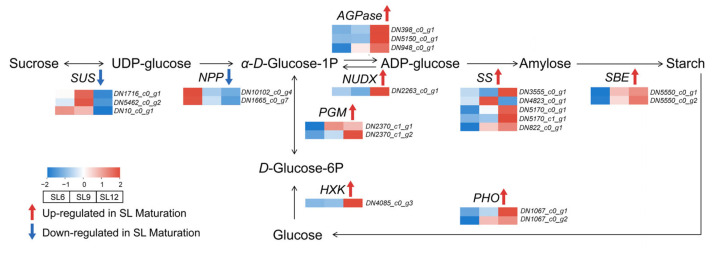
Transcript-level changes in starch and sucrose metabolism during tuber maturation in *Sparganium stoloniferum* tubers (SL). A simplified pathway of starch and sucrose metabolism is shown, covering sucrose cleavage, hexose phosphate interconversion, ADP-glucose formation, and starch synthesis and remodeling. Heatmaps show normalized relative transcript abundance of representative genes across SL6, SL9, and SL12. Red indicates higher expression and blue indicates lower expression. The pathway suggests a maturation-associated shift from early soluble-sugar turnover toward late-stage starch synthesis and restructuring. Abbreviations: SUS, sucrose synthase; NPP, nucleotide pyrophosphatase/phosphodiesterase; AGPase, ADP-glucose pyrophosphorylase; NUDX, nudix hydrolase; SS, starch synthase; SBE, starch-branching enzyme; PHO, starch phosphorylase; HXK, hexokinase; PGM, phosphoglucomutase; UDP-glucose, uridine diphosphate glucose; ADP-glucose, adenosine diphosphate glucose.

To further explore the molecular basis underlying the coordinated development of cell walls and starch accumulation, based on previously generated transcriptome data, transcript-guided pathway analyses were performed focusing on carbohydrate metabolism, lipid-related metabolism, and diterpenoid-associated developmental regulation. Pathway analysis revealed extensive changes in starch and sucrose metabolism during tuber maturation (Figure 6; Table S1). In the early stage, genes associated with sucrose turnover, including *sucrose synthase* (*SUS*), and *nucleotide pyrophosphatase/phosphodiesterase (NPP*), tended to decrease as the tuber matured, suggesting that soluble-sugar utilization and rapid sugar interconversion were relatively more active before the mature storage stage. In contrast, multiple genes involved in the conversion of hexose phosphates into starch precursors and starch polymers showed increased expression toward SL12. These genes included *hexokinase (HXK*), *phosphoglucomutase* (*PGM*), *ADP-glucose pyrophosphorylase* (*AGPase*), *nudix hydrolase* (*NUDX*), *starch synthase* (*SS*), *starch-branching enzyme* (*SBE*), and *starch phosphorylase* (*PHO*), indicating reinforcement of the pathway from glucose phosphorylation and glucose-1-phosphate formation to ADP-glucose production and starch assembly. Among these, the increased expression of *AGPase*, *SS*, and *SBE* suggests enhanced capacity for ADP-glucose generation as well as glucan-chain synthesis and branching during maturation, whereas the induction of *PHO* may reflect active starch remodeling in mature storage tissue. Overall, the transcript pattern supports a shift from early soluble-sugar metabolism toward late-stage starch synthesis and restructuring. This trend is consistent with the marked increase in starch granule accumulation and the ordered starch structure observed in mature winter-harvest SL. Consistently, the selected transcripts showed decreased expression of several sucrose-turnover-related genes and increased expression of several starch-synthesis-related genes toward SL12.

### 2.7. Transcript-Level Changes in Fatty Acid Synthesis and Processing During Tuber Maturation

**Figure 7:**
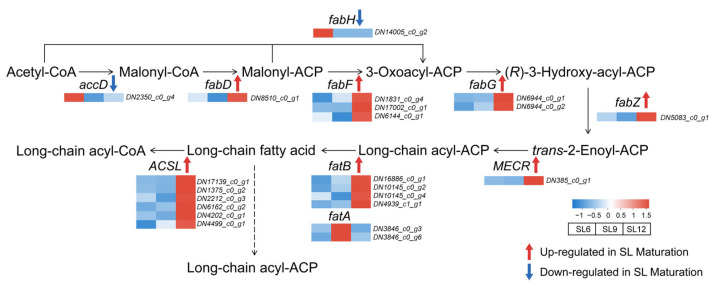
Transcript-level changes in fatty acid synthesis and processing during tuber maturation in *Sparganium stoloniferum* tubers (SL). A simplified fatty acid-related metabolic module is shown, including the conversion from acetyl-CoA to malonyl-CoA, fatty acid chain initiation and elongation, fatty acid release, and long-chain acyl-CoA formation. Heatmaps show normalized relative transcript abundance of representative genes across SL6, SL9, and SL12. Red indicates higher expression and blue indicates lower expression. The pathway highlights stage-dependent changes in genes involved in fatty acid synthesis and processing and suggests selective remodeling of the lipid environment during tuber maturation. Abbreviations: accD, acetyl-CoA carboxylase carboxyltransferase beta subunit; fabH, 3-oxoacyl-[acyl-carrier-protein] synthase III; fabD, malonyl-CoA–acyl carrier protein transacylase; fabF, 3-oxoacyl-[acyl-carrier-protein] synthase II; fabG, 3-oxoacyl-[acyl-carrier-protein] reductase; fabZ, 3-hydroxyacyl-[acyl-carrier-protein] dehydratase; MECR, mitochondrial trans-2-enoyl-CoA reductase; fatB, Acyl-acyl carrier protein thioesterase B; fatA, acyl-acyl carrier protein thioesterase A; ACSL, long-chain acyl-CoA synthetase; ACP, acyl carrier protein; CoA, coenzyme A.

Genes involved in fatty acid synthesis and processing showed coordinated but non-uniform changes during tuber maturation (Figure 7; Table S2). In the early steps of fatty acid synthesis, the transcript level of *acetyl-CoA carboxylase carboxyltransferase beta subunit* (*accD*), which is associated with the conversion from acetyl-CoA to malonyl-CoA, decreased toward SL12. Likewise, *3-oxoacyl-[acyl-carrier-protein] synthase III* (*fabH*), which participates in the initiation of acyl-chain synthesis, also showed a declining trend during maturation. These patterns suggest that some initiation-related reactions were relatively less active in winter-harvest SL than in earlier stages. In contrast, several downstream steps involved in fatty acid chain elongation and modification showed the opposite tendency. The expression of *malonyl-CoA–acyl carrier protein transacylase* (*fabD*), *3-oxoacyl-[acyl-carrier-protein] synthase II* (*fabF*), *3-oxoacyl-[acyl-carrier-protein] reductase* (*fabG*), and *3-hydroxyacyl-[acyl-carrier-protein] dehydratase* (*fabZ*) increased toward SL12, indicating enhanced processing of elongating acyl chains during maturation. In particular, the increased expression of *mitochondrial trans-2-enoyl-CoA reductase* (*MECR*) suggests strengthened reduction steps associated with long-chain acyl-ACP formation in winter-harvest SL. These transcriptional changes indicate that lipid-related metabolism did not change uniformly across the pathway, but instead shifted selectively toward downstream elongation and modification reactions. Additional changes were also observed in genes associated with fatty acid release and activation. *Acyl-acyl carrier protein thioesterase B* (*fatB*) showed increased expression in SL12, whereas *acyl-acyl carrier protein thioesterase A* (*fatA*) displayed a more stage-specific pattern, with relatively higher expression at the intermediate stage rather than a continuous increase toward maturity. Meanwhile, *long-chain acyl-CoA synthetase* (*ACSL*) was clearly upregulated in winter-harvest SL, suggesting enhanced conversion of long-chain fatty acids into long-chain acyl-CoA forms. Overall, fatty acid-related transcripts showed pathway-specific changes during tuber maturation, with several downstream elongation- and activation-related genes showing higher expression in SL12.

### 2.8. Transcript-Level Changes in Gibberellin-Related Pathways During Tuber Maturation

**Figure 8:**
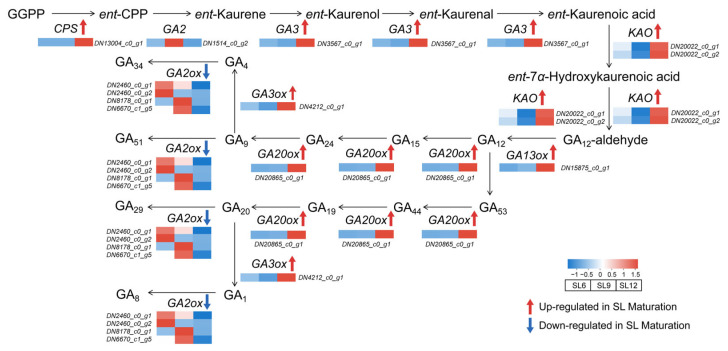
Transcript-level changes in gibberellin biosynthesis during the transition from growth to storage maturation in *Sparganium stoloniferum* tubers (SL). A simplified diterpenoid pathway is shown, extending from GGPP to *ent*-copalyl diphosphate, *ent*-kaurene, and downstream gibberellin biosynthetic branches. Heatmaps show normalized relative transcript abundance of representative genes across SL6, SL9, and SL12. Red indicates higher expression and blue indicates lower expression. Abbreviations: CPS, ent-copalyl diphosphate synthase; GA2, ent-kaur-16-ene synthase; GA3, ent-kaurene oxidase; KAO, kaurenoic acid oxidase; GA13ox, gibberellin 13-oxidase; GA20ox, gibberellin 20-oxidase; GA3ox, gibberellin 3-oxidase; GA2ox, gibberellin 2-oxidase; GGPP, geranylgeranyl diphosphate; *ent*-CPP, *ent*-copalyl diphosphate; GA, gibberellin.

The diterpenoid pathway showed maturation-associated transcript-level changes that were concentrated in the gibberellin-related branch (Figure 8; Table S3). Upstream steps from geranylgeranyl diphosphate (GGPP) to *ent*-copalyl diphosphate and *ent*-kaurene showed increased transcript abundance, as indicated by the induction of *ent-copalyl diphosphate synthase* (*CPS*) and related early biosynthetic nodes. Downstream oxidation steps leading from *ent*-kaurene-derived intermediates toward gibberellin precursors also showed increased transcript levels, including genes associated with *kaurenoic acid oxidase* (*KAO*) and downstream gibberellin-biosynthetic nodes. In parallel, genes involved in the formation of gibberellin intermediates and precursors, such as *gibberellin 13-oxidase* (*GA13ox*), *gibberellin 20-oxidase* (*GA20ox*), and *gibberellin 3-oxidase* (*GA3ox*), showed predominantly higher transcript abundance in SL12. By contrast, the *gibberellin 2-oxidase* (*GA2ox*) branch, which is associated with gibberellin deactivation and catabolite formation, showed a decreasing trend across multiple nodes. These results show that selected gibberellin-biosynthesis-related transcripts increased toward SL12, whereas several GA2ox-related transcripts decreased. Since endogenous gibberellin (GA) levels and functional validation were not examined in this study, these patterns are interpreted as transcript-level associations with tuber maturation.

### 2.9. RT–qPCR Validation of Representative Transcriptomic Patterns

**Figure 9:**
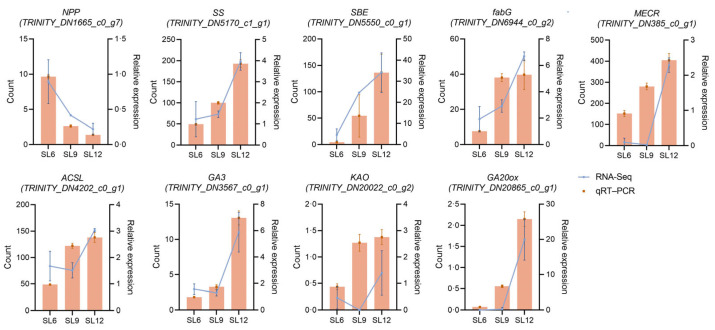
Reverse transcription quantitative polymerase chain reaction (RT–qPCR) validation of representative transcriptomic patterns in *Sparganium stoloniferum* tubers (SL) during development. Representative DEGs from starch and sucrose metabolism, fatty acid metabolism, and gibberellin-related pathways were selected for RT–qPCR validation across SL6, SL9, and SL12. The selected genes included *NPP*, *SS*, *SBE*, *fabG*, *MECR*, *ACSL*, *GA3*, *KAO*, and *GA20ox*. Orange bars represent relative expression levels determined by RT–qPCR, and blue lines represent RNA-seq-derived expression levels. RT–qPCR data were normalized using *ACTIN3* as the internal reference gene and calculated using the 2^−ΔΔCt^ method. Data are presented as mean ± SD from three biological replicates and three technical replicates. SL6, SL9, and SL12 represent tubers collected in June, September, and December, respectively. Abbreviations: NPP, nucleotide pyrophosphatase/phosphodiesterase; SS, starch synthase; SBE, starch-branching enzyme; fabG, 3-oxoacyl-[acyl-carrier-protein] reductase; MECR, mitochondrial trans-2-enoyl-CoA reductase; ACSL, long-chain acyl-CoA synthetase; GA3, ent-kaurene oxidase; KAO, kaurenoic acid oxidase; GA20ox, gibberellin 20-oxidase.

To validate the transcript-guided pathway analysis, nine representative differentially expressed genes (DEGs) from starch and sucrose metabolism, fatty acid metabolism, and gibberellin-related pathways were selected for reverse transcription quantitative polymerase chain reaction (RT–qPCR) analysis (Figure 9). Supplementary phylogenetic trees of the candidate genes are provided in Figures S2–S10 and were considered together with KEGG annotation and developmental expression profiles during the selection of genes for RT–qPCR validation. These genes included *NPP*, *SS*, *SBE*, *fabG*, *MECR*, *ACSL*, *GA3*, *KAO*, and *GA20ox*. The RT–qPCR results were generally consistent with the RNA-seq-derived expression trends. *NPP* showed a decreasing expression pattern from SL6 to SL12, whereas *SS* and *SBE* showed increased expression toward SL12. Fatty acid-related genes, including *fabG*, *MECR*, and *ACSL*, also showed higher expression at later developmental stages, particularly in SL12. Similarly, gibberellin-related genes, including *GA3*, *KAO*, and *GA20ox*, were generally more highly expressed in mature SL. Minor stage-specific differences were observed between the RT–qPCR and RNA-seq profiles for some genes, but the overall expression patterns were broadly consistent. These results support the reliability of the representative transcript-level patterns used for pathway interpretation.

## 3. Discussion

Previous phenotypic and mechanical measurements from the same developmental sample set showed that SL increased in size, weight, and rigidity during maturation [8]. The present study extends those observations by showing that winter-harvest SL is characterized by reduced moisture content, increased tuber density, greater parenchyma cell density, thickened multilamellar cell walls, and abundant starch deposition. These features indicate that the traditional quality trait of heavy and firm winter-harvest SL is associated with a combined structural phenotype rather than with a single compositional factor. Histological, confocal, SEM, and TEM observations consistently showed progressive tissue compaction, inward cell-wall thickening, and dense intracellular starch accumulation. Therefore, winter-harvest quality in SL is best interpreted as the outcome of coordinated tissue consolidation, wall maturation, starch deposition, and developmental transition.

Cell-wall thickening appears to provide an important structural basis for the increasing firmness of mature SL. Plant cell-wall mechanics depend on the organization and interaction of cellulose, hemicellulose, pectin, and other wall-associated polymers, rather than on the abundance of a single component alone [16,17,18]. Intercellular adhesion also contributes to tissue coherence and mechanical strength [19]. In this context, the increased relative cell-wall content, multilamellar wall architecture, and compact cellular arrangement observed in winter-harvest SL suggest that wall reinforcement accompanies SL maturation. The spectroscopic results support this interpretation. FTIR and solid-state ^13^C CP/MAS NMR showed that the isolated walls remained predominantly polysaccharide-rich, while subtle changes in fingerprint, aromatic, and methoxy-associated regions became more evident at later stages. These results suggest reinforcement or remodeling of an existing wall scaffold rather than a major compositional transition.

Starch deposition is another major feature of the mature phenotype. Winter-harvest SL contained abundant intracellular starch granules, and isolated SL12 starch showed an A-type crystalline pattern, short-range molecular order, and relatively high gelatinization and pasting temperatures. These properties indicate that starch in mature SL is not only abundant but also structurally organized. The diffraction signal near 20° and the FTIR band near 2850 cm^−1^ may be compatible with amylose–lipid-complex-related organization, but these features alone do not directly confirm the presence or molecular composition of amylose–lipid complexes. This interpretation is consistent with previous studies showing that amylose–lipid complexes can restrict water penetration, reduce starch swelling and solubilization, and influence starch crystallinity, gelatinization, retrogradation, rheological behavior, and thermal stability [13,20,21]. Thus, the mature starch fraction may contribute to intracellular filling and tissue consolidation, but the fine molecular basis of its stability remains to be clarified. Future work using GPC-based starch component analysis, amylopectin chain-length profiling, and direct analysis of starch-bound lipids would help determine how starch molecular organization contributes to winter-harvest firmness.

The transcript-guided pathway analysis provides a plausible developmental context for these structural changes. During maturation, several genes associated with early soluble-sugar turnover decreased, whereas genes involved in hexose phosphorylation, glucose-1-phosphate formation, ADP-glucose production, and starch assembly increased toward SL12. This pattern is consistent with a shift from rapid soluble-sugar interconversion toward storage-reserve formation and wall-associated carbon investment. Because sucrose-derived intermediates can support both starch biosynthesis and cell-wall polysaccharide formation, starch deposition and wall development in winter-harvest SL may represent coordinated outputs of a shared maturation-associated carbon economy.

Lipid-related transcripts showed pathway-specific rather than uniform changes. Initiation-associated genes such as *accD* and *fabH* decreased, whereas several genes involved in downstream chain elongation, reduction, release, and activation increased toward maturity. These changes suggest remodeling of lipid-related metabolism during tuber maturation. Together with the starch physicochemical data, this transcript profile is consistent with the possibility that lipid-related metabolism contributes to the mature starch-storage environment. However, the present study provides transcriptional rather than direct biochemical evidence. Therefore, any relationship between fatty acid metabolism and starch stabilization should be considered hypothetical until starch-bound lipid composition and amylose–lipid complex formation are directly tested.

The gibberellin-related pathway also showed maturation-associated transcript changes. Selected gibberellin-biosynthesis-related transcripts increased toward SL12, whereas several *GA2ox*-related transcripts decreased. These patterns suggest that GA-associated metabolism may be linked to the transition from active tuber growth to storage maturation. Studies in other storage organs indicate that gibberellin can influence secondary growth, vascular differentiation, or storage-root development, but its effects are strongly context dependent [22,23]. Because endogenous GA levels and functional validation were not examined here, the observed GA-related changes should be interpreted as transcript-level associations with tuber maturation rather than direct evidence of hormonal regulation.

Overall, the data support a model in which winter-harvest quality in *S. stoloniferum* is associated with coordinated maturation of the cell-wall framework and starch storage matrix. Reinforced multilayered walls may provide a mechanical framework, whereas abundant and relatively stable starch may enhance intracellular filling and tissue consolidation. Transcript-level changes in carbohydrate metabolism, lipid-related metabolism, and gibberellin-associated pathways provide additional evidence for a coordinated maturation program. More cautiously, these structural features may contribute to the persistence and harvestable quality of mature underground organs, but their roles in overwintering, defense, and commercial quality require further experimental evaluation.

## 4. Materials and Methods

### 4.1. Phenotypic, Histological, Ultrastructural, and Apparent Amylose-Content Analyses of SL at Different Growth Stages

Tissues were cut into approximately 5 mm segments and fixed in formalin–acetic acid–alcohol for 24 h. The samples were dehydrated through a graded ethanol series (70–100%), cleared in xylene, embedded in paraffin, and sectioned at 5 μm. After deparaffinization, the sections were stained with PAS–Naphthol Yellow S and observed using a Zeiss Axio Scope A1 microscope (Zeiss, Oberkochen, Germany). Stele parenchyma cell density and starch distribution were quantified using ImageJ (v1.53e), and cell-wall thickness was measured using ZEN software (v3.9.101.04000). For apparent amylose-content determination, dried SL samples were analyzed using an amylose content assay kit (#BC4260, Solarbio, Beijing, China) according to the manufacturer’s instructions. For moisture content, parenchyma cell density, starch granule density, relative cell-wall content, and apparent amylose content, three biological replicates were analyzed for each developmental stage. For parenchyma cell-wall thickness, tuber density, and tuber volume, twenty independent tubers were analyzed for each developmental stage. Statistical analyses were performed using GraphPad Prism (v9.0.0). Data are presented as mean ± SD, and statistical significance was assessed by one-way ANOVA followed by Tukey’s multiple-comparisons test, with *p* < 0.05 considered significant.

For further characterization, LSCM, SEM, and TEM were performed. For LSCM, the sections were stained with calcofluor white and hydroxynaphthol blue and examined using a TCS SP8 confocal microscope (Leica, Wetzlar, Germany), with detection at 490, 570, and 660 nm. For SEM, the samples were fixed in 2.5% glutaraldehyde, washed with 0.1 M phosphate buffer, post-fixed in 1% osmium tetroxide, dehydrated through graded ethanol (30–95%), treated with ethanol/isopentyl acetate, freeze-dried, fractured, sputter-coated, and imaged using an SU8100 field-emission SEM (Hitachi, Tokyo, Japan) at 3 kV. For TEM, dehydrated samples were infiltrated with resin–acetone mixtures, embedded in pure resin, polymerized at 70 °C, and sectioned to 70 nm using an EM UC7 ultramicrotome (Leica, Wetzlar, Germany). The sections were stained with lead citrate and uranyl acetate and examined using an HT7700 TEM (Hitachi, Tokyo, Japan) at 80 kV.

### 4.2. Extraction of SL Cell Walls at Different Growth Stages

Dried SL were milled and passed through a 60-mesh sieve. Approximately 1 g sample was suspended in 2 mL of 5 mM Na_2_S_2_O_5_ containing 1.5% sodium dodecyl sulfate (SDS) and 15 μL n-octanol, and homogenized on ice for 10 min. The homogenate was centrifuged at 10,000× *g* for 10 min at 4 °C, and the pellet was washed twice with 3 mM Na_2_S_2_O_5_ containing 0.5% SDS, with centrifugation at 10,000× *g* for 10 min at 4 °C after each wash. The pellet was then frozen, ground, resuspended in 1.5 mL of 3 mM Na_2_S_2_O_5_ containing 0.5% SDS and 30 μL n-octanoic acid, and homogenized at 4 °C for 4 h until no intact cells were visible under the microscope.

After centrifugation at 10,000× *g* for 10 min at 4 °C, the insoluble material was extracted with phenol–acetic acid–water (PAW; 4:2:1, *w*/*v*/*v*) at 40 °C for 6 h, centrifuged at 4000× *g* for 10 min at 4 °C, and re-extracted overnight to obtain crude cell-wall material. The crude pellet was then treated twice with DMSO/LiBr at 80 °C overnight. The residue was washed with deionized water until the iodine test was negative, washed twice with absolute ethanol, and dried. Three biological replicates were analyzed.

### 4.3. Fourier Transform Infrared Spectroscopy of SL Cell Walls

CW from SL6, SL9, and SL12 were mixed with dried KBr at a ratio of 1:100 (*w*/*w*) and pressed into pellets under 2 tons of pressure. FTIR spectra were recorded using a Nicolet iS5 FTIR spectrometer (Thermo Fisher, Waltham, MA, USA) over the range of 4000–400 cm^−1^ with 16 scans at a resolution of 4 cm^−1^. KBr was used as the background, and automatic background correction was applied. Six biological replicates were measured. Raw spectra were baseline-corrected and smoothed using OMNIC software (v8.2.0.387), and final plots were generated using OriginPro 2021 (v9.8.0.200).

### 4.4. Solid-State Nuclear Magnetic Resonance Analysis of SL Cell Walls

Approximately 300 mg of cell-wall material from SL6, SL9, and SL12 was analyzed using a Bruker Avance Neo 400WB solid-state NMR spectrometer (Bruker, Rheinstetten, Germany). Solid-state ^13^C CP/MAS NMR spectra were collected in H_2_O/D_2_O with 32 scans and an acquisition time of 0.0259 s. The acquisition temperatures for 13C were 332.0 K for SL6, 324.2 K for SL9, and 324.0 K for SL12. Spectra were processed using MestReNova (v14.0.0).

### 4.5. Preparation of SL Starch

Approximately 300 g of SL12 powder was suspended in 800 mL of distilled water and homogenized for 30 s using a blender (Midea, Foshan, China). The resulting slurry was filtered through a 200-mesh sieve, and the filtrate was allowed to settle at room temperature for 24 h. The precipitate was resuspended in distilled water, and the settling procedure was repeated twice. The final sediment was dried at 40 °C to a constant weight, milled, and passed through a 65-mesh sieve, and stored in a desiccator until analysis.

### 4.6. Differential Scanning Calorimetry of SL Starch

Approximately 3 mg starch sample from SL12 was mixed with 10 μL of distilled water and equilibrated overnight. DSC measurements were performed using a DSC-200F3 differential scanning calorimeter (Netzsch, Selb, Germany) over a temperature range of 20–200 °C at a heating rate of 10 °C/min. The Onset (T_o_), peak (T_p_), and conclusion (T_c_) temperatures, together with the enthalpy change (ΔH) were recorded. Measurements were performed in triplicate.

### 4.7. Rapid Visco Analysis of SL Starch

Pasting properties were measured using an RVA 4500 analyzer (Perten, Stockholm, Sweden). A 2 g starch sample from SL12 was dispersed in 20 mL of deionized water and subjected to the following temperature program: equilibration at 50 °C for 1 min, heating to 95 °C at 12 °C/min and holding for 2.5 min, followed by cooling to 50 °C at 12 °C/min and holding for 2 min. Peak, trough, and final viscosities, as well as breakdown, setback, and pasting temperature, were recorded.

### 4.8. X-Ray Diffraction of SL Starch

X-ray diffraction patterns were collected using a D8 Advance diffractometer (Bruker, Karlsruhe, Germany) with Cu-Kα radiation over a scanning range of 5–90° at a scanning speed of 2°/min. Relative crystallinity was calculated using JADE software (v6.0).

### 4.9. Fourier Transform Infrared Spectroscopy of SL Starch

SL12 starch samples were mixed with dried KBr at a ratio of 1:20 (*w*/*w*), and FTIR analysis was performed as described in Section 4.3.

### 4.10. Raman Spectroscopy of SL Starch

Raman spectra of dried SL12 starch were acquired using an HR Evolution Raman spectrometer (Horiba, Kyoto, Japan) with 785 nm excitation over the range of 3300–100 cm^−1^. Spectra were collected from three different positions and processed using OriginPro 2021 (v9.8.0.200). The full width at half maximum (FWHM) of the band at 480 cm^−1^ was calculated.

### 4.11. Integrated Analysis of Physicochemical Properties, Transcriptomics, and RT–qPCR Validation

The transcriptomic dataset used in this study was obtained from the NCBI Sequence Read Archive under accession number PRJNA987745. Processed expression data and differential-expression information were used as previously described in the corresponding published study [8]. For the present work, genes related to starch and sucrose metabolism, fatty acid synthesis and processing, and gibberellin biosynthesis were extracted according to KEGG annotation and developmental expression patterns. Nine representative genes, including *NPP*, *SS*, *SBE*, *fabG*, *MECR*, *ACSL*, *GA3*, *KAO*, and *GA20ox* were selected based on KEGG annotation, developmental expression patterns, and supplementary phylogenetic information. Homologous nucleotide sequences of the candidate genes were aligned using MUSCLE in MEGA (v11.0.13). Phylogenetic trees were constructed using the Neighbor-Joining method with the Maximum Composite Likelihood model. Bootstrap analysis was performed with 1000 replicates, and gaps or missing data were treated using pairwise deletion. To validate the transcriptomic patterns, representative differentially expressed genes from starch and sucrose metabolism, fatty acid metabolism, and gibberellin-related pathways were selected for RT–qPCR analysis. These genes included *NPP*, *SS*, *SBE*, *fabG*, *MECR*, *ACSL*, *GA3*, *KAO*, and *GA20ox*. Total RNA was extracted from frozen SL6, SL9 and SL12 samples using a plant total RNA isolation kit (#RC401, Vazyme, Nanjing, China) according to the manufacturer’s instructions. RNA concentration and purity were assessed using a Nano-300 NanoDrop (Allsheng, Hangzhou, China). RNA integrity was evaluated by agarose gel electrophoresis. First-strand cDNA was synthesized using HiScript III 1st strand cDNA synthesis kit (#R312, Vazyme, Nanjing, China). RT–qPCR assays were conducted following established protocols [8], and relative transcript levels were calculated using the 2^−ΔΔCt^ method [24]. *ACTIN3* (*TRINITY_DN501_c1_g1*, NCBI: XP_010912922.1) was used as the internal reference gene. Primer sequences are provided in Table S4.

## Data Availability

The transcriptomic dataset analyzed in this study is available in the NCBI Sequence Read Archive under accession number PRJNA987745. The data that support the findings of this study are available from the corresponding author upon reasonable request.

## Supplementary Materials

The following supporting information can be downloaded at: https://www.mdpi.com/article/10.3390/ijms27104566/s1.
