# Supplementary material for: Coordinated Cell-Wall and Starch Maturation Is Associated with Winter-Harvest Quality in Sparganium stoloniferum Tubers
Source: Int J Mol Sci. 2026 May 19;27(10):4566. doi: 10.3390/ijms27104566 (PMC13207313; doi:10.3390/ijms27104566)
Supplement: Supplementary file 1 [file ijms-27-04566-s001.zip › ijms-4300911-supplementary.pdf]

Coordinated Cell-Wall and Starch Maturation Is Associated with Winter-Harvest Quality in *Sparganium stoloniferum* Tubers

Xilong Qian [1,2,3], Maoqi Pan [3], Jingying Zhang [3], Qinan Liu [4], Fan Yang [1,2,3], Chanchan Liu [1,2,3], Mengru Sang [1,2,3,*] and Qinan Wu [1,2,3,*]

[1] National Key Laboratory on Technologies for Chinese Medicine Pharmaceutical Process Control and Intelligent Manufacture, Nanjing University of Chinese Medicine, Nanjing 210023, China; qxl@njucm.edu.cn (X.Q.); 300586@njucm.edu.cn (F.Y); liuchanchan@njucm.edu.cn (C.L.)
[2] Jiangsu Collaborative Innovation Center of Chinese Medicinal Resources Industrialization, Nanjing University of Chinese Medicine, Nanjing 210023, China
[3] School of Pharmacy, Nanjing University of Chinese Medicine, Nanjing 210023, China; pan12@njucm.edu.cn (M.P.); zjy20030823@163.com (J.Z.);
[4] Nanjing Institute for Food and Drug Control, Nanjing 211198, China; liuqinan0728@163.com (Q.L.)
* Correspondence: sangmengru@njucm.edu.cn (M.S.); wuqn@njucm.edu.cn (Q.W.)

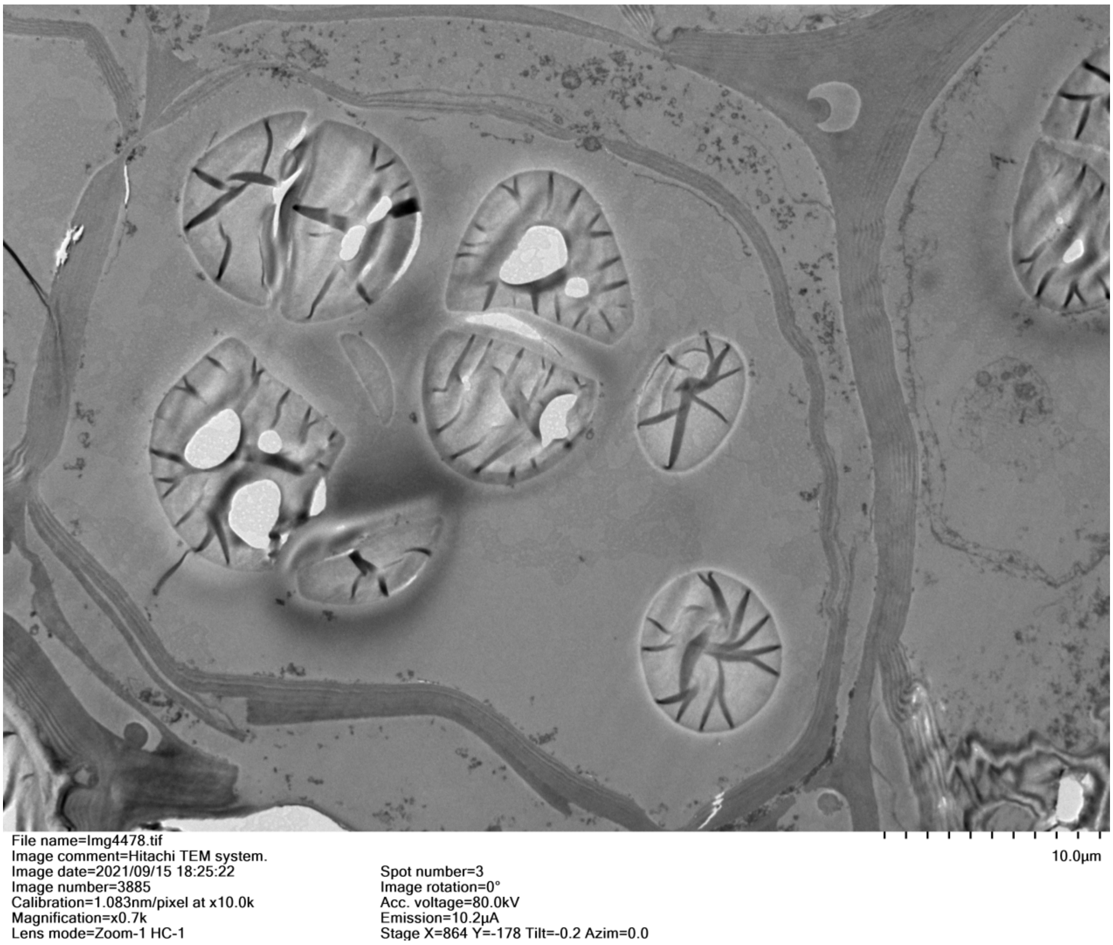

Figure S1. Transmission electron microscopy observation of starch-rich parenchyma cells in mature *Sparganium stoloniferum* tubers (SL12), scale bar = 10.0 μm.

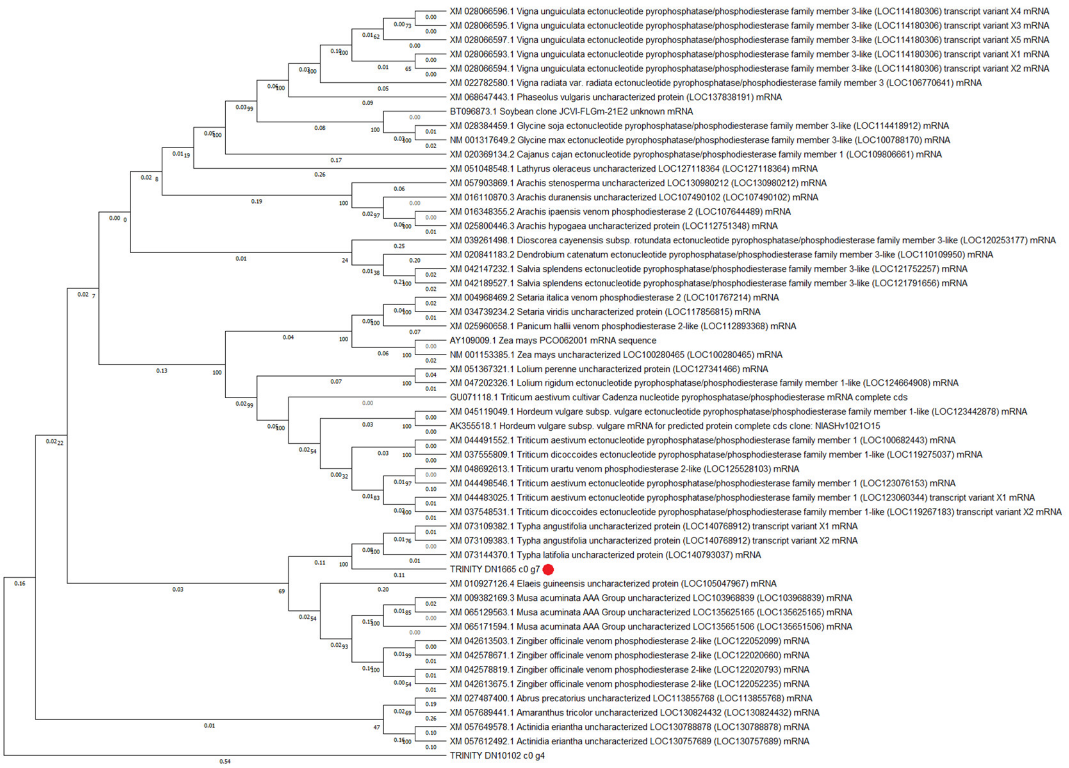

Figure S2. Phylogenetic analysis of *nucleotide pyrophosphatase/phosphodiesterase* (*NPP*) homologs. The tree was constructed using homologous transcript-derived nucleotide sequences from *Sparganium stoloniferum* and other plant species to support the annotation of the *NPP* transcript selected for RT–qPCR validation. The selected *S. stoloniferum* transcript is marked in the tree. This phylogenetic analysis was used as supplementary annotation support together with KEGG annotation, transcript abundance, and primer specificity.

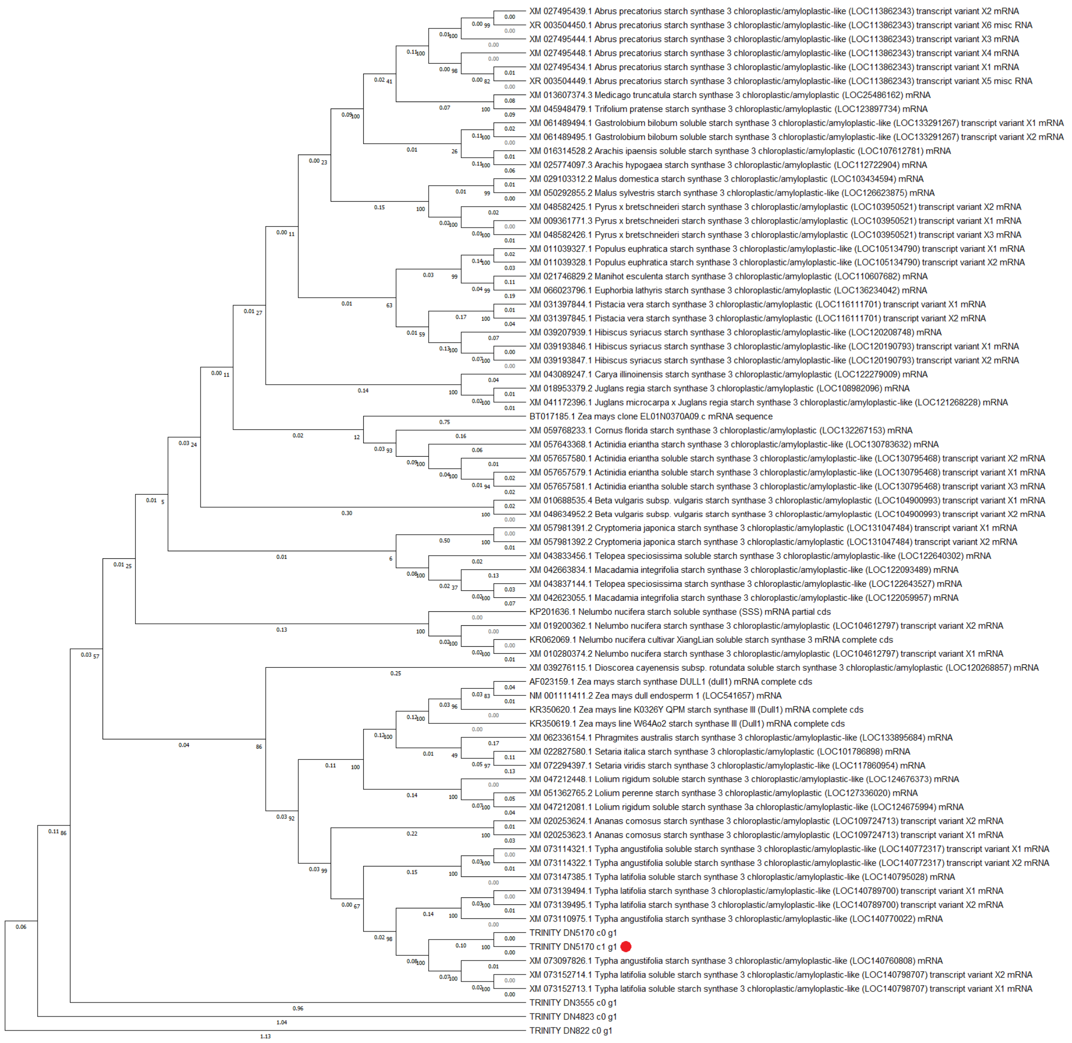

Figure S3. Phylogenetic analysis of *starch synthase* (*SS*) homologs. The tree was constructed using homologous transcript-derived nucleotide sequences from *Sparganium stoloniferum* and other plant species to support the annotation of the *SS* transcript selected for RT–qPCR validation. The selected *S. stoloniferum* transcript is marked in the tree. This phylogenetic analysis was used as supplementary annotation support together with KEGG annotation, transcript abundance, and primer specificity.

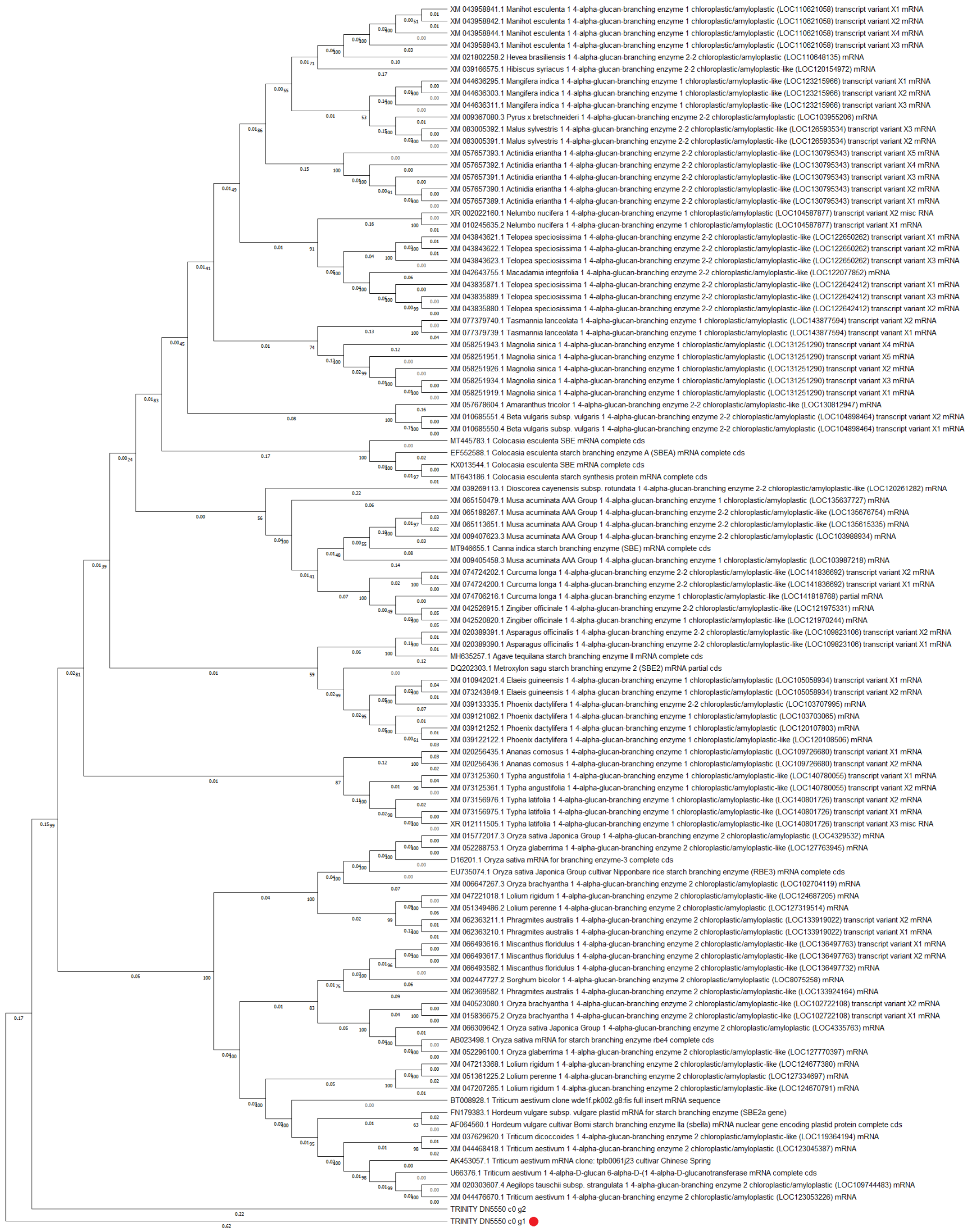

Figure S4. Phylogenetic analysis of *starch-branching enzyme* (*SBE*) homologs. The phylogenetic tree was constructed using homologous transcript-derived nucleotide sequences from *Sparganium stoloniferum* and other plant species. Two *S. stoloniferum SBE*-like transcripts, *TRINITY_DN5550_c0_g1* and *TRINITY_DN5550_c0_g2*, were included in the analysis. Both transcripts were positioned outside the major *SBE* clades, suggesting that they may represent divergent or partial *SBE*-like transcripts rather than definitive *SBE* orthologs. Among them, *TRINITY_DN5550_c0_g1* contained a more complete predicted coding region and showed higher transcript abundance than *TRINITY_DN5550_c0_g2*. Therefore, it was selected for RT–qPCR validation and is marked in the tree. This phylogenetic analysis was used as supplementary annotation evidence together with KEGG annotation, predicted CDS integrity, transcript abundance, and primer specificity.

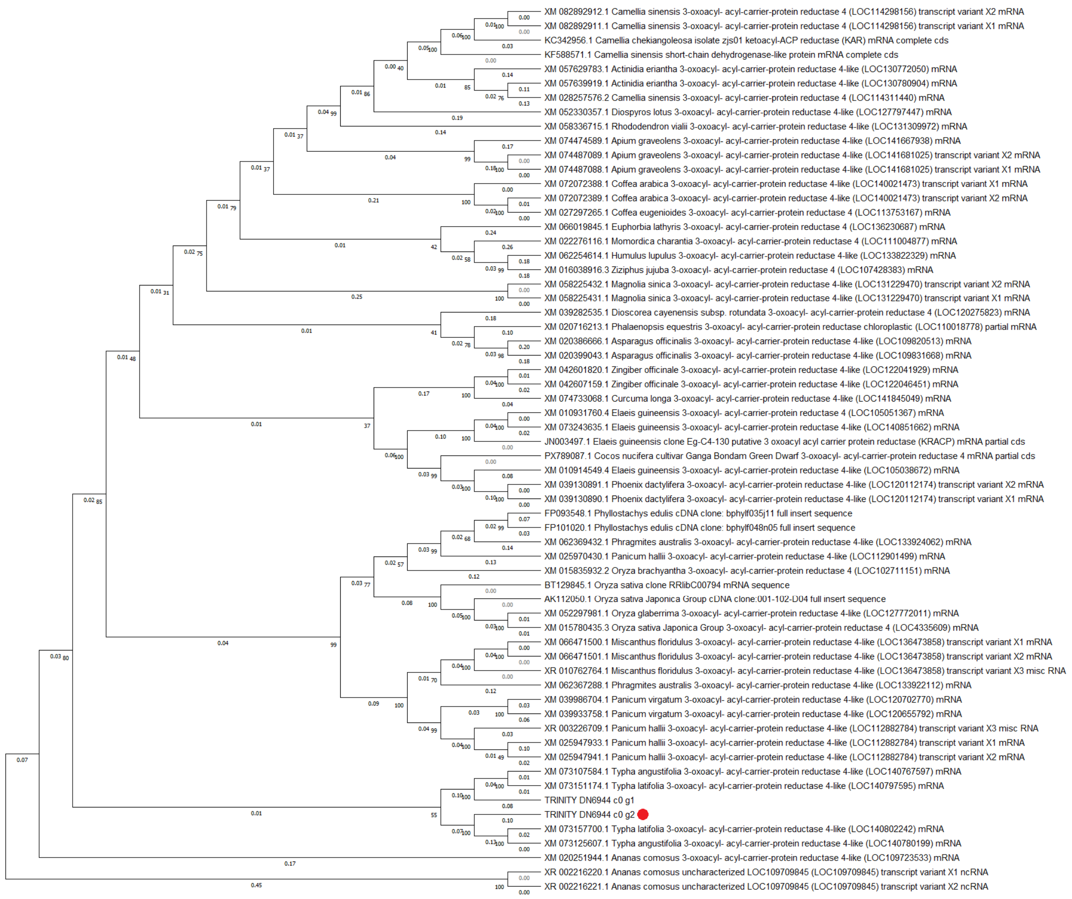

Figure S5. Phylogenetic analysis of *3-oxoacyl-[acyl-carrier-protein] reductase* (*fabG*) homologs. The tree was constructed using homologous transcript-derived nucleotide sequences from *Sparganium stoloniferum* and other plant species to support the annotation of the *fabG* transcript selected for RT–qPCR validation. The selected *S. stoloniferum* transcript is marked in the tree. This phylogenetic analysis was used as supplementary annotation support together with KEGG annotation, transcript abundance, and primer specificity.

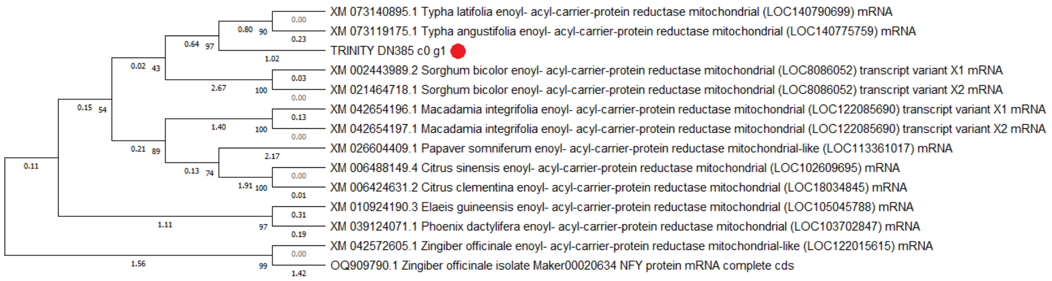

Figure S6. Phylogenetic analysis of *mitochondrial trans-2-enoyl-CoA reductase* (*MECR*) homologs. The tree was constructed using homologous transcript-derived nucleotide sequences from *Sparganium stoloniferum* and other plant species to support the annotation of the *MECR* transcript selected for RT–qPCR validation. The selected *S. stoloniferum* transcript is marked in the tree. This phylogenetic analysis was used as supplementary annotation support together with KEGG annotation, transcript abundance, and primer specificity.

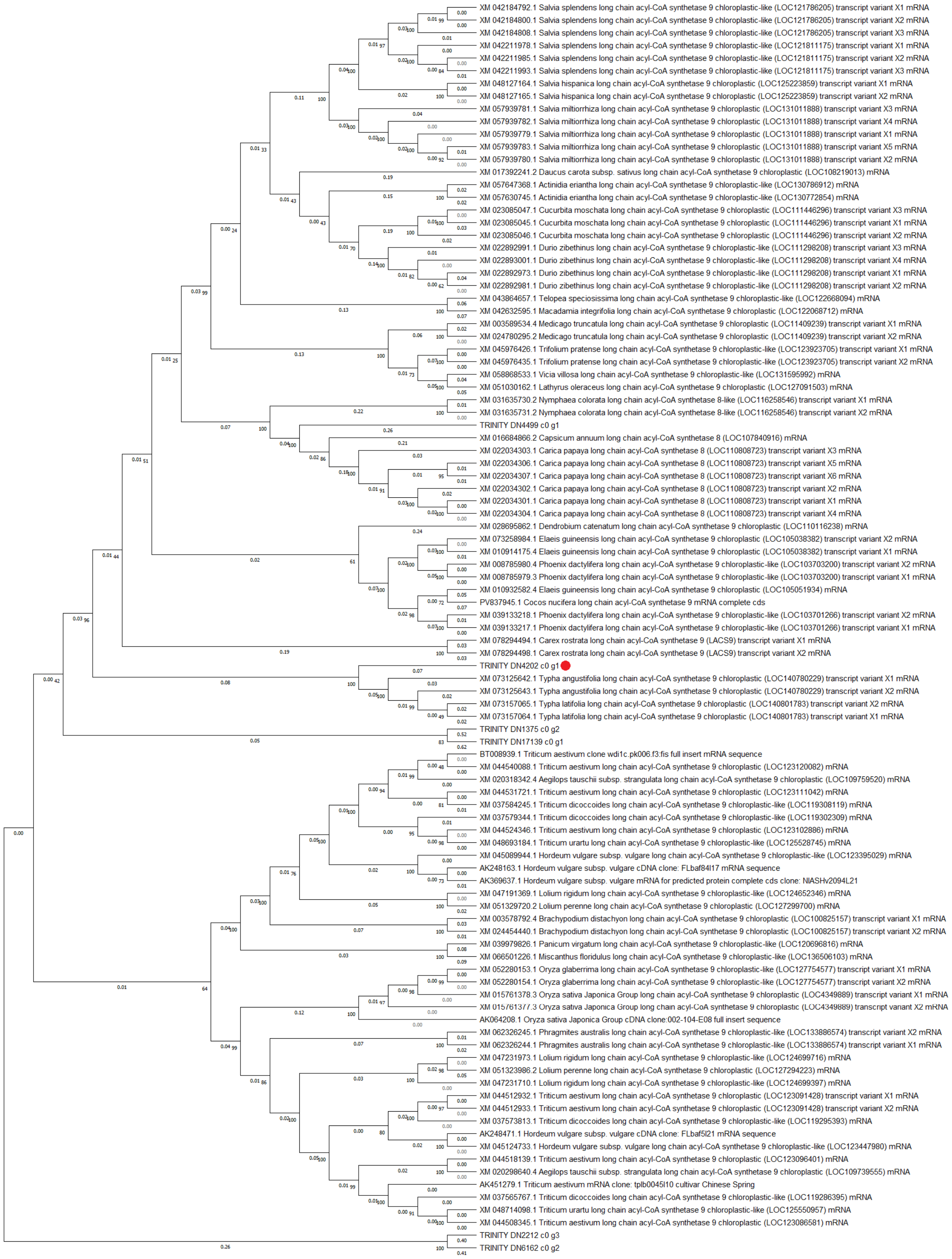

Figure S7. Phylogenetic analysis of *long-chain acyl-CoA synthetase* (ACSL) homologs. The tree was constructed using homologous transcript-derived nucleotide sequences from *Sparganium stoloniferum* and other plant species to support the annotation of the *ACSL* transcript selected for RT–qPCR validation. The selected *S. stoloniferum* transcript is marked in the tree. This phylogenetic analysis was used as supplementary annotation support together with KEGG annotation, transcript abundance, and primer specificity.

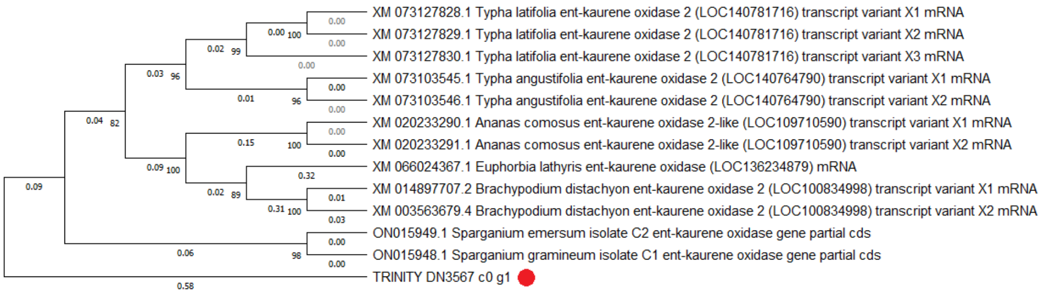

Figure S8. Phylogenetic analysis of *ent-kaurene oxidase* (*GA3*) homologs. The phylogenetic tree was constructed using homologous transcript-derived nucleotide sequences from *Sparganium stoloniferum* and other plant species. The selected *S. stoloniferum* transcript, *TRINITY_DN3567_c0_g1*, is indicated in the tree. Although this transcript was positioned outside the main *ent-kaurene oxidase* clade and did not cluster closely with the *Typha* or *Sparganium* reference sequences, it was retained as an *ent-kaurene oxidase*-like candidate based on transcriptome annotation, KEGG annotation, transcript abundance, predicted sequence features, and primer specificity. Therefore, this phylogenetic analysis was used as supplementary annotation evidence rather than definitive proof of a specific orthologous relationship.

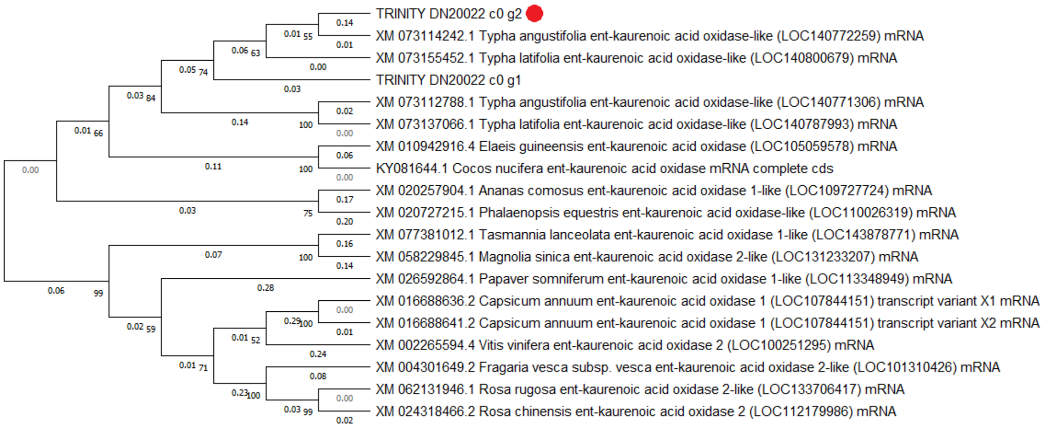

Figure S9. Phylogenetic analysis of *kaurenoic acid oxidase* (*KAO*) homologs. The tree was constructed using homologous transcript-derived nucleotide sequences from *Sparganium stoloniferum* and other plant species to support the annotation of the *KAO* transcript selected for RT–qPCR validation. The selected *S. stoloniferum* transcript is marked in the tree. This phylogenetic analysis was used as supplementary annotation support together with KEGG annotation, transcript abundance, and primer specificity.

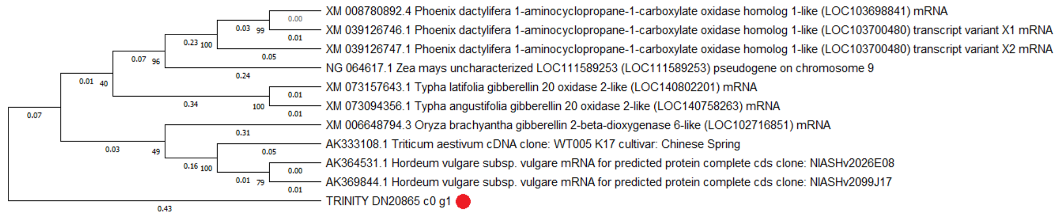

Figure S10. Phylogenetic analysis of *gibberellin 20-oxidase* (*GA20ox*) homologs. The phylogenetic tree was constructed using homologous transcript sequences from *Sparganium stoloniferum* and other plant species. Because *GA20ox* belongs to the *2-oxoglutarate-dependent dioxygenase (2ODD)* family, related *2ODD* sequences were included to evaluate the broad homologous relationship of the candidate transcript. The selected *S. stoloniferum* transcript, *TRINITY_DN20865_c0_g1*, is indicated in the tree. Although this transcript did not cluster closely with the *Typha GA20ox2*-like clade, it was retained as a *GA20ox*-like candidate based on transcriptome annotation, predicted coding sequence integrity, transcript abundance, and primer specificity. Therefore, this phylogenetic analysis was used as supplementary annotation evidence rather than definitive proof of a specific *GA20ox* ortholog.

Table S1 Representative genes used for the starch and sucrose metabolism pathway analysis in *Sparganium stoloniferum* tubers, including gene names, Trinity IDs, and relative transcript abundance in SL6, SL9, and SL12

Gene	Trinity ID	SL6_1	SL6_2	SL6_3	SL9_1	SL9_2	SL9_3	SL12_1	SL12_2	SL12_3
SUS	TRINITY_DN1716_c0_g1	41.84	209.66	107.39	254.11	173.78	250.76	4.33	3.92	6.44
SUS	TRINITY_DN5462_c0_g2	78.39	83.81	46.72	135.75	286.42	122.13	6.21	1.93	7.28
SUS	TRINITY_DN10_c0_g1	2600.06	1038.20	1835.64	1833.20	1289.86	1828.79	472.05	565.14	533.51
NPP	TRINITY_DN10102_c0_g4	20.77	7.14	16.84	3.35	4.42	3.32	1.33	2.34	1.64
NPP	TRINITY_DN1665_c0_g7	12.11	8.81	5.89	4.19	4.02	4.23	2.88	2.45	1.30
AGPase	TRINITY_DN398_c0_g1	98.65	4.51	6.52	435.26	260.74	434.59	3170.47	3407.23	2704.26
AGPase	TRINITY_DN5150_c0_g1	0.20	1.63	0.64	1.20	1.18	0.85	26.46	29.99	21.56
AGPase	TRINITY_DN948_c0_g1	89.04	42.63	79.91	335.70	216.01	339.01	470.57	500.84	359.44
NUDX	TRINITY_DN2263_c0_g1	1.55	12.99	8.09	4.68	3.80	4.44	27.53	26.50	25.21
SS	TRINITY_DN3555_c0_g1	4.43	4.95	9.16	2.09	1.01	1.87	15.68	21.08	24.44
SS	TRINITY_DN4823_c0_g1	103.87	16.03	124.86	293.77	106.01	290.07	16.13	14.73	9.02
SS	TRINITY_DN5170_c0_g1	1.07	1.10	4.34	7.64	4.36	8.03	11.98	14.45	8.91
SS	TRINITY_DN5170_c1_g1	15.05	73.24	95.72	77.29	64.20	78.03	222.19	188.95	183.58
SS	TRINITY_DN822_c0_g1	3.85	2.01	1.86	48.93	49.63	50.44	60.81	72.20	59.03
SBE	TRINITY_DN5550_c0_g1	15.47	9.20	31.14	99.16	98.26	99.02	109.87	179.32	119.93
SBE	TRINITY_DN5550_c0_g2	0.40	0.00	0.00	0.00	0.00	0.00	2.64	3.04	3.46
PHO	TRINITY_DN1067_c0_g1	0.38	0.00	0.22	2.33	0.91	2.73	5.51	11.83	6.99
PHO	TRINITY_DN1067_c0_g2	170.76	19.75	56.22	880.66	721.79	897.60	720.42	1423.35	782.36
HXK	TRINITY_DN4085_c0_g3	0.69	11.60	0.91	4.16	7.38	4.43	27.43	31.55	23.90
PGM	TRINITY_DN2370_c1_g1	15.83	5.80	9.46	120.62	41.90	116.63	74.60	91.95	76.18
PGM	TRINITY_DN2370_c1_g2	0.00	0.12	0.00	1.16	0.00	0.36	1.58	1.84	1.97

Table S2 Representative genes used for the fatty acid synthesis and processing pathway analysis in *Sparganium stoloniferum* tubers, including gene names, Trinity IDs, and relative transcript abundance in SL6, SL9, and SL12

Gene	Trinity ID	SL6_1	SL6_2	SL6_3	SL9_1	SL9_2	SL9_3	SL12_1	SL12_2	SL12_3
accD	TRINITY_DN2350_c0_g4	1.01	2.72	0.86	0.20	0.15	0.20	0.50	0.58	0.51
fabD	TRINITY_DN8510_c0_g1	10.95	20.44	17.40	15.89	11.86	14.41	18.13	21.19	20.87
fabF	TRINITY_DN1831_c0_g4	19.92	15.69	27.48	47.38	31.59	46.09	78.59	87.24	72.53
fabF	TRINITY_DN17002_c0_g1	2.51	9.31	9.38	5.84	6.15	8.13	26.21	26.00	26.09
fabF	TRINITY_DN6144_c0_g1	8.16	6.93	9.36	7.42	3.65	7.08	10.29	12.91	10.19
fabG	TRINITY_DN6944_c0_g1	9.40	5.27	15.66	12.09	6.87	12.63	27.48	24.32	21.11
fabG	TRINITY_DN6944_c0_g2	12.12	8.80	22.49	24.47	17.86	23.39	47.50	52.46	50.71
fabH	TRINITY_DN14005_c0_g2	9.10	15.64	11.44	7.03	3.54	8.46	4.91	7.05	7.20
fabZ	TRINITY_DN5083_c0_g1	32.31	21.03	25.90	21.77	11.07	23.97	54.99	51.60	53.22
MECR	TRINITY_DN385_c0_g1	4.61	5.11	38.79	5.01	2.95	3.92	342.91	392.85	409.15
fatB	TRINITY_DN16886_c0_g1	0.00	0.00	0.00	0.54	0.16	0.42	1.41	1.28	1.71
fatB	TRINITY_DN10145_c0_g2	0.00	0.09	0.00	0.58	0.00	0.42	1.03	1.95	1.90
fatB	TRINITY_DN10145_c0_g4	1.50	2.82	4.08	1.94	2.32	2.52	3.65	3.97	3.79
fatB	TRINITY_DN4939_c1_g1	0.00	0.00	0.00	1.63	0.00	0.00	7.26	6.34	3.68
fatA	TRINITY_DN3846_c0_g3	70.34	37.90	53.14	222.93	268.20	223.83	33.14	30.46	33.55
fatA	TRINITY_DN3846_c0_g6	0.00	0.00	0.61	3.48	8.47	5.01	0.89	0.83	0.98
ACSL	TRINITY_DN17139_c0_g1	2.75	11.97	9.03	8.30	6.18	8.30	30.28	25.67	26.18
ACSL	TRINITY_DN1375_c0_g2	0.00	0.21	0.05	0.07	0.07	0.00	1.06	1.07	0.93
ACSL	TRINITY_DN2212_c0_g3	54.25	88.78	68.92	54.71	54.11	54.65	127.40	112.39	118.18
ACSL	TRINITY_DN6162_c0_g2	26.68	61.98	24.96	34.08	45.68	38.51	47.75	54.75	57.00
ACSL	TRINITY_DN4202_c0_g1	55.73	112.12	83.58	66.13	92.50	68.77	149.02	151.13	155.12
ACSL	TRINITY_DN4499_c0_g1	15.66	54.03	81.75	51.60	60.80	55.35	73.61	65.22	52.94

Table S3 Representative genes used for the diterpenoid and gibberellin biosynthesis pathway analysis in *Sparganium stoloniferum* tubers, including gene names, Trinity IDs, and relative transcript abundance in SL6, SL9, and SL12

Gene	Trinity ID	SL6_1	SL6_2	SL6_3	SL9_1	SL9_2	SL9_3	SL12_1	SL12_2	SL12_3
CPS	TRINITY_DN13004_c0_g1	0	0	0	0	0	0	2.06	0	3.14
GA2	TRINITY_DN1514_c0_g2	0.81	0.96	2.63	10.87	8.67	12.71	0.52	0.31	0.25
GA3	TRINITY_DN3567_c0_g1	2.25	2.81	3.73	2.43	1.97	2.82	14.27	9.59	9.24
KAO	TRINITY_DN20022_c0_g1	0	0.43	0.66	0.14	0	0.14	1.03	0.22	0.82
KAO	TRINITY_DN20022_c0_g2	0	0.27	0.41	0	0	0	1.84	0.27	0
GA13ox	TRINITY_DN15875_c0_g1	0	0	0	0.23	0	0	1.37	0.69	1.62
GA20ox	TRINITY_DN20865_c0_g1	0	0	0	0.07	0	0	0.7	3.7	0.57
GA3ox	TRINITY_DN4212_c0_g1	21.48	16.21	24.44	21.52	19.28	19.8	23.79	21.23	25.42
GA2ox	TRINITY_DN2460_c0_g1	5.21	60.35	1.51	1.35	0.22	2.06	0	0.16	0
GA2ox	TRINITY_DN2460_c0_g2	3.14	3.85	7.47	56.68	73.48	53.46	0	0.15	0.14
GA2ox	TRINITY_DN8178_c0_g1	52.11	12.12	24.25	61.7	33.65	59.51	4.53	7.67	4.98
GA2ox	TRINITY_DN6670_c1_g5	0	0.3	0.53	0.15	0.34	0.07	0	0	0

Table S4 Primer sequences used for RT–qPCR.

Gene	Forward primer	Reverse primer
TRINITY_DN501_c1_g1 (ACTIN3)	5′-ACAGCAGGCAAAGGAGTGGCA-3′	5′-TTGAGGTGGCTCGGAAGGTGA-3′
TRINITY_DN6944_c0_g2 (fabG)	5′-TGATATGACTGCCAAACT-3′	5′-TACATTACCATCCCACC-3′
TRINITY_DN385_c0_g1 (MECR)	5′-GGGTCATACCATCTCCTCCTTCCTT-3′	5′-CTGTAACTGTAGCAGCATACTCCAT-3′
TRINITY_DN4202_c0_g1 (ACSL)	5′-ATGGTCGTCGGTTAGCT-3′	5′-CGCCTCCAGAAAGTAGA-3′
TRINITY_DN1665_c0_g7 (NPP)	5′-ATGGCATCATCAACAACC-3′	5′-AATCCCTGCTCCACAAC-3′
TRINITY_DN5170_c1_g1 (SS)	5′-GCCATTTCTCCTCATCTTT-3′	5′-AGCAGCCTTCTTACCTTCA-3′
TRINITY_DN5550_c0_g1 (SBE)	5′-CGCTGATAAGTGGATTG-3′	5′-CTCTGCCCACCTTCTAT-3′
TRINITY_DN3567_c0_g1 (GA3)	5′-TCCATCCCAGTCCTTTTTGTTCA-3′	5′-CTCCTGCTCCAATCATCCCTCTA-3′
TRINITY_DN20022_c0_g2 (KAO)	5′-CGGTTGTGATGGTGGCG-3′	5′-TCGGGATCGTTGGATTTGA-3′
TRINITY_DN20865_c0_g1 (GA20ox)	5′-CTGCCCTAGAGCCGACAA-3′	5′-CCACCATCTCCGACCAAG-3′
